# Supplementary material for: Regulation of Protein Structural Changes by Incorporation of a Small-Molecule Linker
Source: Int J Mol Sci. 2018 Nov 22;19(12):3714. doi: 10.3390/ijms19123714 (PMC6321067; doi:10.3390/ijms19123714)
Supplement: Supplementary file 1 [file ijms-19-03714-s001.pdf]

Supplementary Materials

Regulation of Protein Structural Changes by Incorporation of a Small-Molecule Linker

Youngmin Kim [1, 2], Cheolhee Yang[1, 2], Tae Wu Kim [1, 2], Kamatchi Thamilselvan [1, 2], Yongkwan Kim [1, 2], and Hyotcherl Ihee [1, 2,*]

[1] Department of Chemistry and KI for the BioCentury, Korea Advanced Institute of Science and Technology (KAIST), Daejeon 34141, Republic of Korea; youngmin.kim.k@gmail.com (Y.K.); eangel04@gmail.com (C.Y.); ktwk6660@gmail.com (T.W.K.); nanostar.shruthi@gmail.com (K.T.); anglerlord@gmail.com (Y.K.)

[2] Center for Nanomaterials and Chemical Reactions, Institute of Basic Science (IBS), Daejeon 34141, Republic of Korea

[*] Correspondence: hyotcherl.ihee@kaist.ac.kr; Tel.: +82-42-350-2844

Received: date; Accepted: date; Published: date

Preparation of Photoactive Yellow Protein (PYP)

Samples were purified using a previously published method [1,2]. Cysteine mutations were introduced in the reconstructed apo-PYP at the 7th and 91st amino acids positions in the pQE80 vector, which was over-expressed in *Escherichia coli* BL21 (DE3) by isopropyl β-D-1-thiogalactopyranoside (IPTG) induction. After harvesting and sonication of the mutated PYP from *E. coli*, the resulting apo-PYP was incubated with a large amount of the linker molecule and Tris(2-carboxyethyl)phosphine (TCEP), and then reconstituted with *p*-coumaric anhydride and purified through Ni affinity and ion-exchange chromatography. We used enterokinase as a protease to remove the tag without any residual amino acids. The purified protein was further diluted in 20 mM Tris buffer (pH 7.0, 20 mM NaCl).

Matrix-Assisted Laser Desorption/Ionization Time-Of-Flight (MALDI-TOF). Molecular weights of PYP and bleached PYP (PYP with the chromophore removed) were measured using a MALDI-TOF mass spectrometer (Bruker Autoflex III, Bruker Daltonics Inc., Germany). Sinapinic acid was used as a matrix material for measurement. The laser source for MALDI-TOF was the third harmonic of an Nd:YAG laser (355 nm), and the repetition rate was 1 Hz.

Circular Dichroism. To confirm the secondary structure of the G7C-M91C mutant PYP and SML-PYP, circular dichroism (CD) spectra were measured using a CD spectrometer (Jasco-815, JASCO Inc., Japan) with a 2-mm quartz cuvette at room temperature. The spectral window ranged from 190 nm to 260 nm at 0.2-nm intervals. The baseline was measured with the same buffer in the same cuvette and subtracted.

Experiment-Restrained Rigid-Body molecular dynamics (MD) Simulation. This method was

almost identical to that used in a previous study [3,4]. Here, a crystal structure (2PHY) was used as a starting point. The structures were divided into a number of rigid bodies comprising several amino acids. The rigid bodies were allowed to move under the influence of a chemical and χ^2 force field. Because the atomic structure within a rigid body is constrained to that of the crystal or nuclear magnetic resonance (NMR) structure, the force field within the rigid body is not considered; however, Van der Waals interactions between rigid bodies and N-C bond-length corrections between rigid bodies are included in the chemical force field. The χ^2 force field is introduced to drive the molecular structure generated by MD simulations toward a structure that yields a different scattering curve that matches the experimental difference scattering curve. Thus, the total potential (U) on the rigid bodies has the Van der Waals term U_{LJ}, the χ^2 term, and the bond correction term B_{ij} as follows:

$$U = c_1 U_{LJ} + c_2\chi^2 + c_3 B_{ij} \tag{1}$$

where c_1, c_2, and c_3 are weighting parameters used to scale the magnitude of the three terms. U_{LJ} and χ^2, which define the agreement between the experimental data and theoretical values, are calculated as follows; scaling factor c_s and reduced units are used in this calculation.

$$U_{LJ} = \sum_{i=1}^{N}\sum_{j\neq i}^{N} 4\left(\frac{\sigma^{12}}{r_{ij}^{12}} - \frac{\sigma^{6}}{r_{ij}^{6}}\right) \tag{2}$$

$$\chi^2 = \sum_{q=1}^{N_q}\left(\frac{I_{\exp}(q) - c_s I_{cal}(q)}{\sigma_q}\right)^2 \tag{3}$$

The force field is the gradient of the total potential; therefore, the total force acting on the i-th particle among the total number of particles N is as follows:

$$
\begin{aligned}
\mathbf{f}_i &= -\nabla_i U \\
&= 24c_1 \sum_{j\neq i}^{N} \left(2\frac{\sigma^{12}}{r_{ij}^{13}} - \frac{\sigma^{6}}{r_{ij}^{7}} \right) \\
&+ 2c_2 c_s \sum_{q=1}^{N_q} \left(\frac{I_{\exp}(q) - c_s I_{cal}(q)}{\sigma_q^2} \right) \sum_{j\neq i}^{N} f_i^s(q) f_j^s(q) \left(\cos(qr_{ij}) - \frac{\sin(qr_{ij})}{qr_{ij}} \right) \frac{\mathbf{r}_{ij}}{r_{ij}^2}
\end{aligned}
$$

(4)

In the L-J potential, σ is defined as the r value where the corresponding potential is zero and $\mathbf{r}_{ij}$ (= $\mathbf{r}_i - \mathbf{r}_j$) is the distance between particle *i* and particle *j*. If the distance between the two particles is smaller than σ, repulsion increases steeply. We defined the following two types of σ values for the L-J potential between rigid bodies: for the N-C bond between two rigid bodies consisting of helices, $\sigma_{N\text{-}C}$ = 1.28 Å, and for the atoms between two rigid bodies, $\sigma_{a\text{-}a}$ = 1.2 Å. The role of the bond correction term B_{ij} is to simply maintain the N-C bond distance between rigid bodies at the initial value ($B_{ij} = \sigma_{N\text{-}C} - 1.28$ Å). Once the total forces between rigid bodies were determined, MD simulations were run on the basis of Newtonian equations. The coordinate of the center of mass (COM) of the rigid body was as follows:

$$
\mathbf{R} = \frac{\sum m_i \mathbf{r}_i}{M},
$$

(5)

where m_i and $\mathbf{r}_i$ are the mass and position of the *i*-th atom of the rigid body, respectively, and *M* is the mass of the rigid body ($M = \sum_i m_i$).

The COM moved translationally, and the relative rotational motion with respect to the COM was determined according to the relative position ($\mathbf{r}_i' = \mathbf{r}_i - \mathbf{R}$) of the *i*-th atom with reference to **R**, $\mathbf{r}_i'$. At each step of the MD simulation, **R,** $\mathbf{r}_i$, and $\mathbf{r}_i'$ were updated. The equation for the translational motion of the COM is as follows:

$$\ddot{\mathbf{R}} = \frac{\mathbf{F}}{M} \,. \tag{6}$$

where $\mathbf{F} = \sum_i \mathbf{f}_i$ is the total force on the rigid body and the sum of $\mathbf{f}_i$ acting on the i-th atom within the rigid body. The total torque acting on each rigid body with respect to the COM is as follows:

$$\mathbf{N} = \sum_i \mathbf{r}_i' \times \mathbf{f}_i \,. \tag{7}$$

The velocity of $\mathbf{r}_i'$ relative to $\mathbf{R}$ is as follows:

$$\dot{\mathbf{r}}_i' = \boldsymbol{\omega} \times \mathbf{r}_i' \tag{8}$$

where $\boldsymbol{\omega}$ is the angular velocity with respect to the COM of the rigid body. The moment of inertia with respect to the COM is as follows:

$$\mathbf{I} = \begin{bmatrix} \sum m_i (y_i'^2 + z_i'^2) & -\sum m_i x_i'^2 y_i'^2 & -\sum m_i x_i'^2 z_i'^2 \\ -\sum m_i x_i'^2 y_i'^2 & \sum m_i (x_i'^2 + z_i'^2) & -\sum m_i y_i'^2 z_i'^2 \\ -\sum m_i x_i'^2 z_i'^2 & -\sum m_i y_i'^2 z_i'^2 & \sum m_i (x_i'^2 + y_i'^2) \end{bmatrix} \tag{9}$$

The rotational equation of the motion of a rigid body around $\mathbf{R}$ can be expressed as[4]

$$\dot{\boldsymbol{\omega}} = \mathrm{I}^{-1} \cdot (\mathrm{N} - \boldsymbol{\omega} \times (\mathrm{I} \cdot \boldsymbol{\omega})) \tag{10}$$

Eq. (5) updates $\mathbf{R}$ and, combining eqns. (7)–(10), updates $\mathbf{r}_i'$.

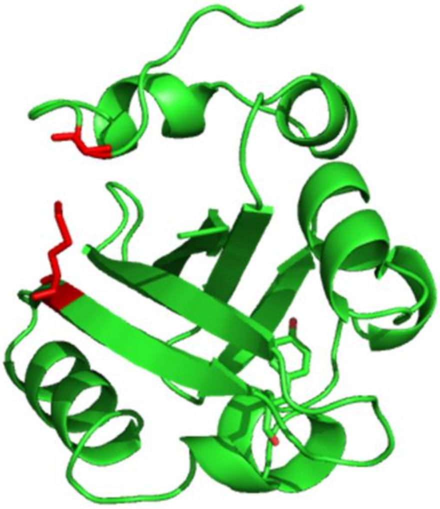

Figure S1. The 7th (glycine) and 91st (methionine) positions of PYP (PDB ID: 2PHY) were mutated to cysteines. The residues are shown in red.

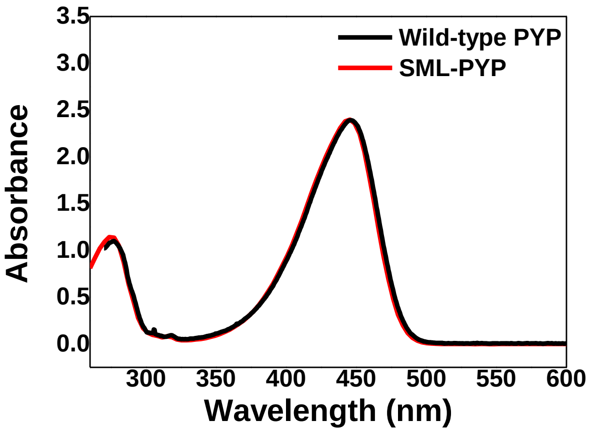

Figure S2. UV-vis spectra of wild-type PYP (black) and BM(PEG)2-incorporated PYP (SML-PYP, red).

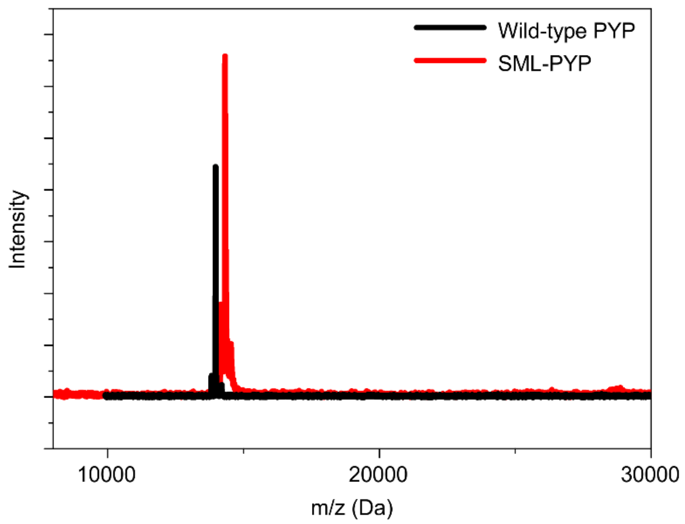

Figure S3. MALDI/TOF result for wild-type PYP (black, 14.02 kDa) and BM(PEG)$_2$-incorporated PYP (SML-PYP) (red, 14.36 kDa). The molecular weight (M/W) of PYP was 14.02 kDa and BM(PEG)2 was 308.29 Da, and the sum of these two values (14.33 kDa) was almost equal to the MALDI/TOF result for BM(PEG)$_2$-incorporated PYP.

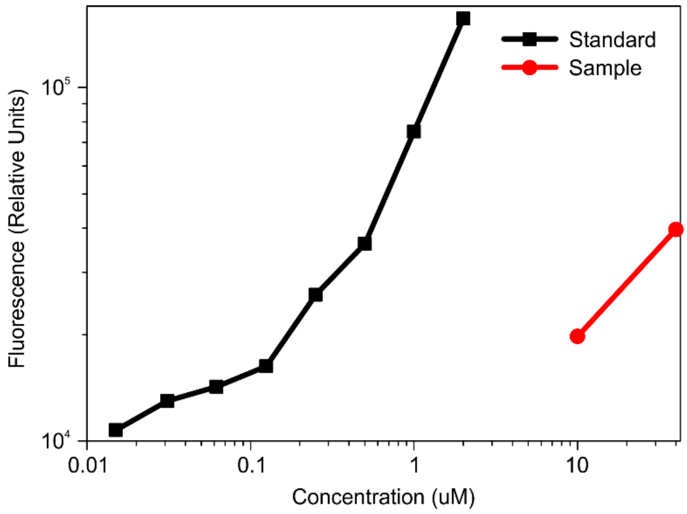

Figure S4. Thiol Fluorescent Detection Kit (Invitrogen™) result. Black indicates the standard solution fluorescence intensity. Red indicates the sample fluorescence intensity (SML-PYP). 10 µM sample (SML-PYP) solution contains only 0.17 µM free thiols.

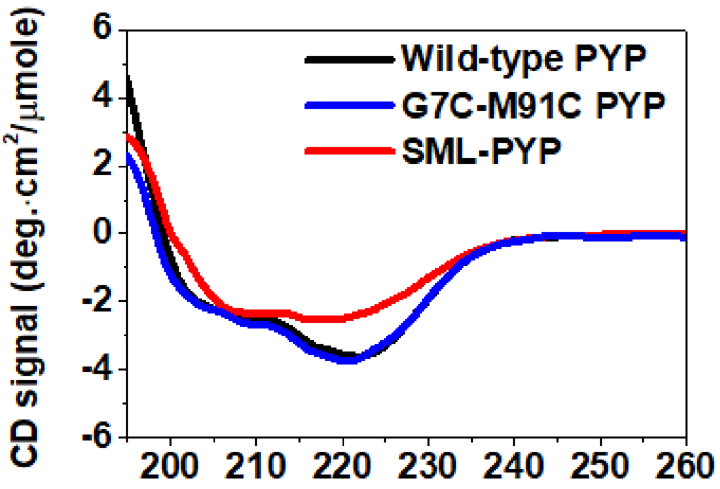

Figure S5. Circular dichroism spectra of wild-type PYP, G7C-M91C mutant, and SML-PYP. Whereas wild-type PYP and mutated PYP show similar CD spectra, the CD spectrum of SML-PYP indicates that its a-helix content is reduced probably due to unfolded N-term region.

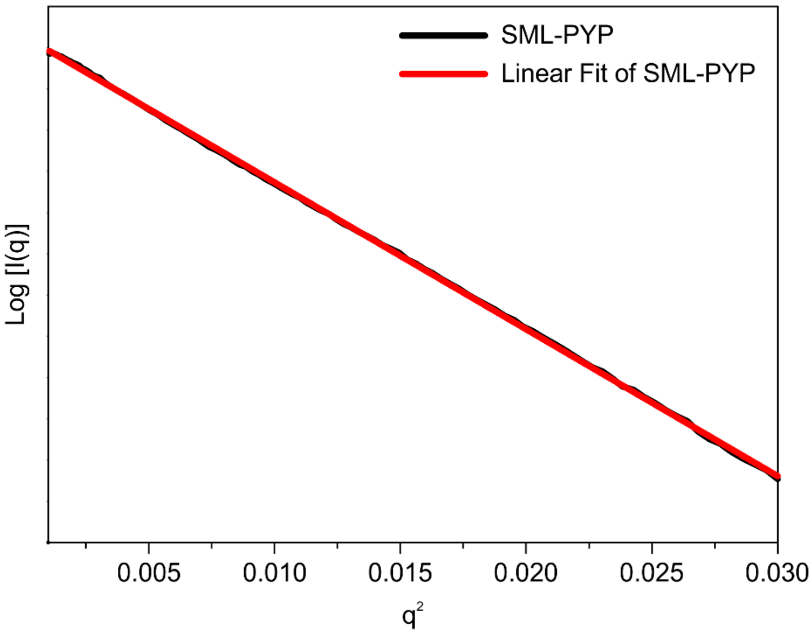

Figure S6. Guinier region of SAXS data for BM(PEG)$_2$-incorporated PYP (SML-PYP) in the dark state. PYP is a highly soluble protein and even after the incorporation of BM(PEG)$_2$ the solubility of the protein was maintained. To avoid any distortion of X-ray scattering curve originated from the aggregated species, the solution was cautiously centrifuged in 10,000 g for 10 min prior to performing the SAXS measurements. The Guinier plot of the original SAXS data shows excellent linearity with an R-square value of 0.9999, confirming the absence of aggregation in the protein solution.

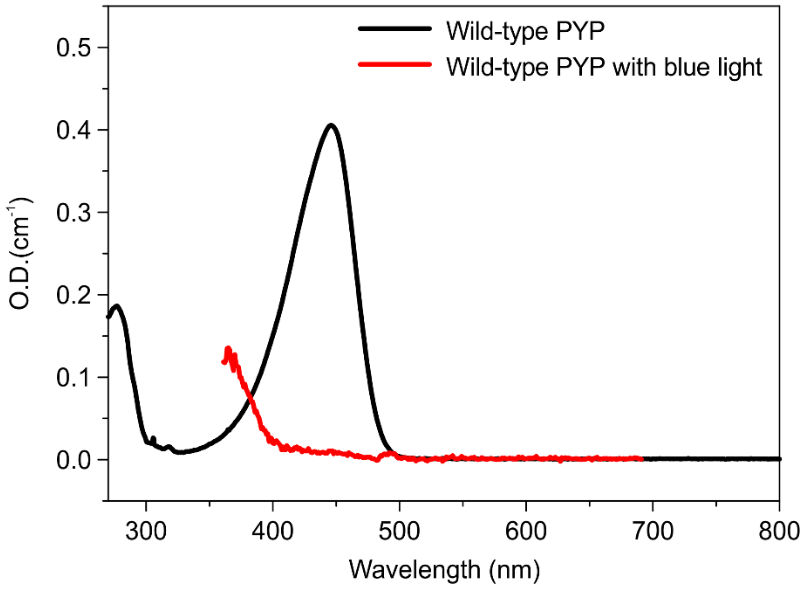

Figure S7. UV-vis spectra of PYP with/without continuous LED illumination. The spectrum with continuous LED illumination barely shows the peak corresponding to pG, indicating that pG has been converted to pB under the used illumination condition. The fraction of pG is estimated to be less than 2.5%. When this spectrum was measured, the concentration of the solution was 10 μM and the volume was 2 mL. For the SAXS measurement, the concentration was 0.71 mM, but the volume (1 μL) was much smaller, thus, the total number of proteins in the SAXS measurement was even smaller than that in the UV-vis measurement. In addition, the same LED source was used with tighter focusing for the SAXS measurement, and thus, the pG fraction under continuous LED illumination is expected be even much smaller than 2.5% in the SAXS measurement.

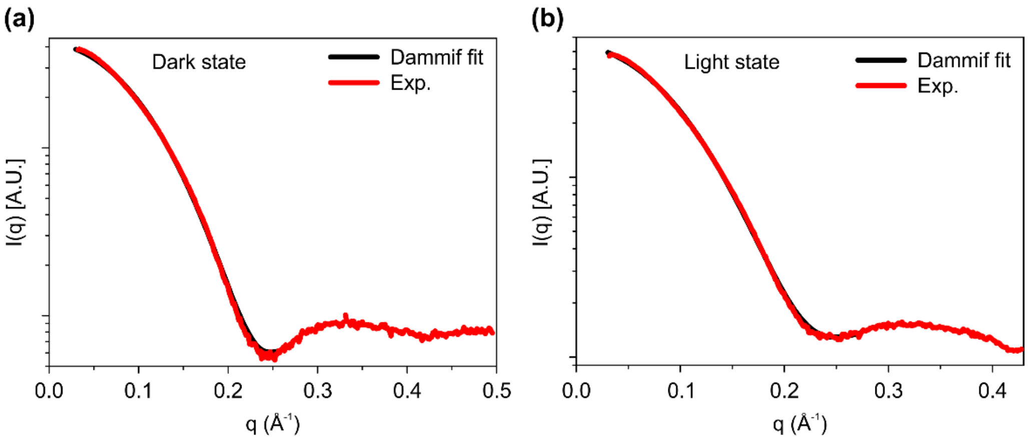

Figure S8. Comparison of DAMMIF-fit curves (black) and the experiment curves (red) from static SAXS data for SML-PYP in dark (a) and light (b) states for low-resolution shape reconstruction. The chi-square values are 3.8 and 2.3 in dark and light state, respectively.

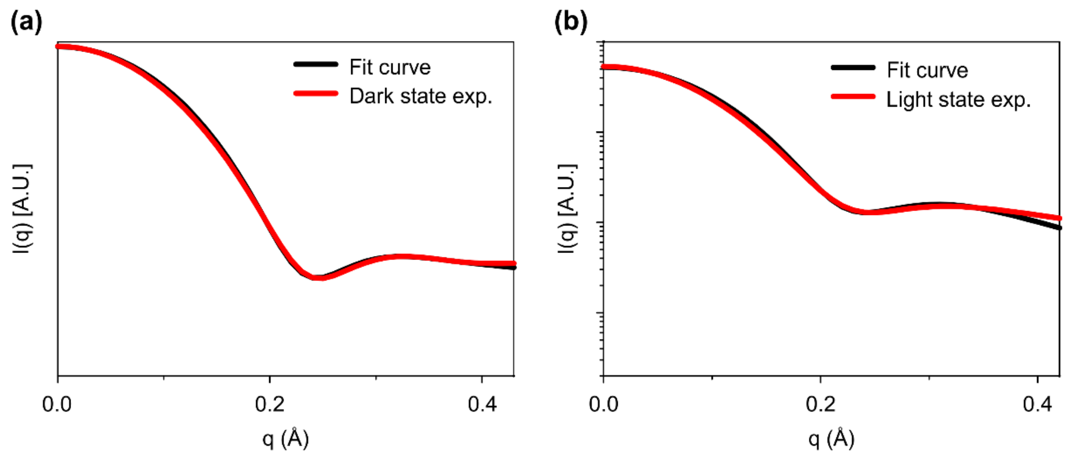

Figure S9. GNOM curve from static SAXS data (red) and the fit curve from experiment-restrained rigid-body molecular dynamics simulation (black) for SML-PYP in dark (a) and light (b) states.

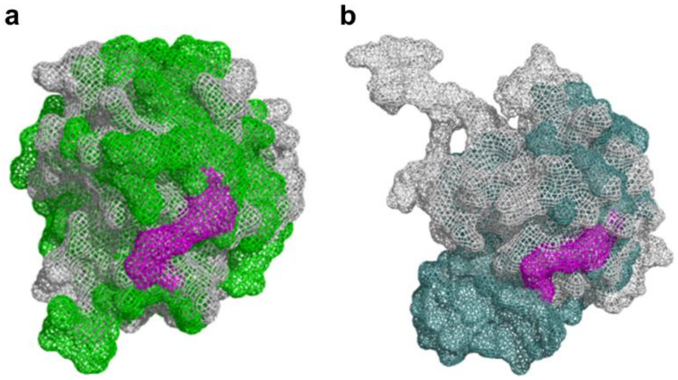

Figure S10. Protein structures refined from the experiment-restrained rigid-body MD simulation and previously reported wild-type PYP and pB$_2$ structure. (a) Comparison of pG structures of SML-PYP and wild-type PYP. The protein conformations color-coded by green and gray colors correspond to pG states of SML-PYP and wild-type PYP, respectively. (b) Comparison of SML-PYP and pB$_2$ structures of wild-type (PDB ID: 2KX6). Blue and gray colors indicate pB$_2$ of SML-PYP and wild-type PYP, respectively. The magenta mesh shows the BM(PEG)$_2$ moiety covalently linked to the 7th and 91st residues in the protein.

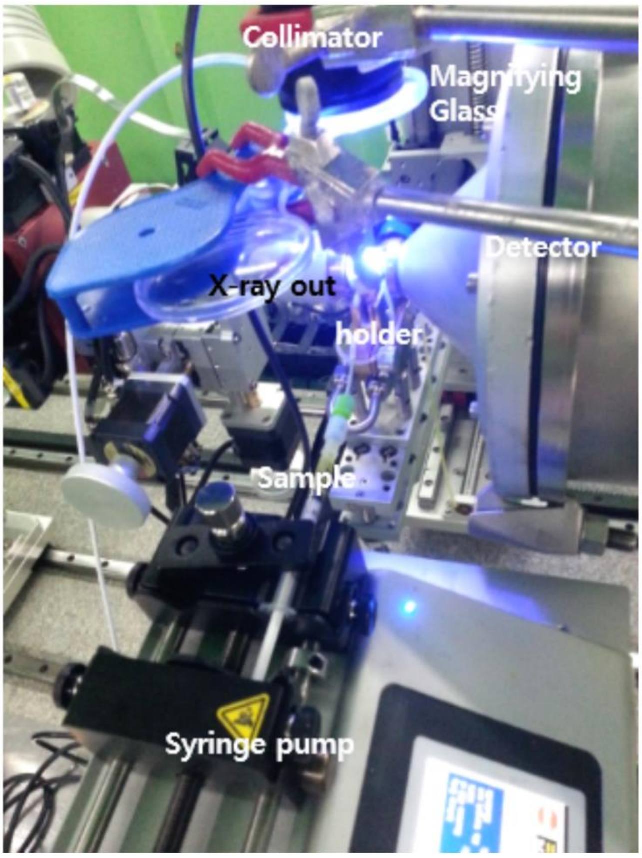

Figure S11. SAXS setup for SML-PYP in the light state. The sample solution in the capillary holder was irradiated by focusing a 445-nm LED light (1000 mW) through a collimator and magnifying glass.

References

1. Imamoto, Y.; Ito, T.; Kataoka, M.; Tokunaga, F. Reconstitution photoactive yellow protein from apoprotein and P-coumaric acid-derivatives. *FEBS Lett* **1995**, *374*, 157-160, DOI: 10.1016/0014-5793(95)01096-W.
2. Kumar, A.; Burns, D.C.; Al-Abdul-Wahid, M.S.; Woolley, G.A. A Circularly permuted photoactive yellow protein as a scaffold for photoswitch design. *Biochemistry* **2013**, *52*, 3320-3331, DOI: 10.1021/bi400018h.
3. Kim, Y.; Ganesan, P.; Jo, J.; Kim, S.O.; Thamilselvan, K.; Ihee, H. Chromophore-removal-induced conformational change in photoactive yellow protein determined through spectroscopic and X-ray solution scattering studies. J. Phys. Chem. B 2018, 122, 4513-4520, DOI: 10.1021/acs.jpcb.8b01768.
4. Ahn, S.; Kim, K.H.; Kim, Y.; Kim, J.; Ihee, H. Protein tertiary structural changes visualized by time-resolved X-ray solution scattering. J. Phy. Chem. B 2009, 113, 13131-13133, DOI: 10.1021/jp906983v.
